# Enhancing Tax Compliance through Coercive and Legitimate Power of Tax Authorities by Concurrently Diminishing or Facilitating Trust in Tax Authorities

**DOI:** 10.1111/lapo.12021

**Published:** 2014-04-29

**Authors:** Eva Hofmann, Katharina Gangl, Erich Kirchler, Jennifer Stark

## Abstract

Both coercion, such as strict auditing and the use of fines, and legitimate procedures, such as assistance by tax authorities, are often discussed as means of enhancing tax compliance. However, the psychological mechanisms that determine the effectiveness of each strategy are not clear. Although highly relevant, there is rare empirical literature examining the effects of both strategies applied in combination. It is assumed that coercion decreases implicit trust in tax authorities, leading to the perception of a hostile antagonistic tax climate and enforced tax compliance. Conversely, it is suggested that legitimate power increases reason-based trust in the tax authorities, leading to the perception of a service climate and eventually to voluntary cooperation. The combination of both strategies is assumed to cause greater levels of intended compliance than each strategy alone. We conducted two experimental studies with convenience samples of 261 taxpayers overall. The studies describe tax authorities as having low or high coercive power (e.g., imposing lenient or severe sanctions) and/or low or high legitimate power (e.g., having nontransparent or transparent procedures). Data analyses provide supportive evidence for the assumptions regarding the impact on intended tax compliance. Coercive power did not reduce implicit trust in tax authorities; however, it had an effect on reason-based trust, interaction climate, and intended tax compliance if applied solely. When wielded in combination with legitimate power, it had no effect.

## I. Introduction

Tax collection is an important endeavor for tax authorities. Essential strategies for increasing tax compliance include deterrence and assistance through legitimated procedures (Gangl et al. 2012; Alm and Torgler 2011; Braithwaite 2003). Taxpayers' trust in authorities, as well as in fellow citizens, fosters honest tax contributions as well (Gangl et al. 2012; Alm and Torgler 2011; Braithwaite 2003). Coercion is based on tax audits and fines if tax evasion is detected (Allingham and Sandmo 1972). Legitimacy is based on transparency, fairness, and participation of tax authorities (Alm et al. 2010; Feld and Frey 2007; Wenzel 2002). Trust is based on social norms (Coleman 2007; Wenzel 2004) or moral suasion (Ariel 2011; Alm and Torgler 2011; Torgler 2004). Existing research indicates that coercion and legitimacy should be applied simultaneously in order to increase tax compliance among citizens (Alm and Torgler 2011; Braithwaite 2003). The combination of these different measures may be more efficient in influencing tax compliance than either measure alone (Gangl et al. 2013). However, whether or not underlying psychological processes, such as the level of trust a taxpayer has in tax authorities provide an explanation for the effectiveness of a combination of strategies has not been explored. Shedding light on these underlying mechanisms is essential to tax researchers and practitioners in order to understand how measures to increase tax compliance work and can be applied most effectively.

The slippery slope framework (Kirchler, Hoelzl, and Wahl 2008) explores the mechanisms used by authorities to influence taxpayer decision making and assure tax compliance. Previous research suggests that trust in tax authorities is influenced by whether power is coercive or legitimate creating different climates and motivations to comply (Gangl et al. 2012). They undertook laboratory experiments that manipulated tax authorities' power and trustworthiness. The studies confirm the assumptions of the slippery slope framework (Kogler et al. 2013; Wahl, Kastlunger, and Kirchler 2010) and offer a more nuanced picture of the mechanisms that affect tax compliance. We find that the combination of high power and high trustworthiness leads to overall higher tax compliance than power or trustworthiness alone. This may be because when power is combined with trustworthiness it is perceived as legitimate expert power that motivates compliance. Thus, how power is implemented may be a key determinant of tax compliance.

This article explores the impact of coercive power and legitimate power on psychological processes and subsequent tax compliance. In the three experiments we conducted, coercive power and legitimate power were manipulated independently and in combination by applying scenarios of fictitious tax authorities. In Study 1, we separately examine (1) the effect of coercive power on trust in authorities and (2) the effect of legitimate power on trust in authorities. The study also examines the climate between tax authorities and taxpayers, and its effect on motivation for taxpayers to comply with the authority. In Study 2, we examine the combined effects of coercive power and legitimate power on the above-mentioned underlying processes and tax compliance. Study 1 serves to confirm the theoretical assumptions on the impact of coercive power and of legitimate power. Study 2 explores the effects of using a combination of coercive and legitimate power since it is the currently predominant practice used by taxation authorities.

In the following part, we present the theoretical background for our work. While the distinction between coercive power and legitimate power is comparatively novel to tax research, it has been a well-established distinction in social psychology for decades. We are confident that the insights of the social psychology literature provide an understanding of the underlying mechanisms that cause the effects of coercive power and legitimate power. Parts III and IV report on the experiments in which we manipulate low versus high levels of coercive power and legitimate power, either separately or in combination. Part V summarizes the results and discusses the findings.

## II. Theoretical Background

### A. Power

Scholars in the field of social psychology have often sought to explicitly distinguish between different qualities of power (Tyler 2006; Turner 2005; French and Raven 1959). Turner (2005) and Tyler (2006), for instance, have assumed that depending on the way power is exercised, two qualities of power can be distinguished: coercive power and legitimate power.

Coercive or harsh power manifests itself through negative and positive reinforcements such as through the imposition of sanctions and the granting of benefits (Raven, Schwarzwald, and Koslowsky 2003). While negative reinforcement, such as fines and imprisonment, are common and well-proven measures used by tax authorities (Becker 2011), positive reinforcement in the form of rewards for honest taxpaying is more unusual. Nevertheless, according to psychological theory of operant conditioning (Skinner 1947), both negative and positive reinforcement provoke tax compliance by penalizing unwanted, and by reinforcing wanted, tax behavior. Field experiments indicate that coercive power, that is, audits and fines, affect tax compliance (Kleven et al. 2011; Hasseldine et al. 2007). This affect is either weak and can be contradictory (Ariel 2011; Blackwell 2010; Slemrod, Blumenthal, and Christian 2001). Laboratory experiments also reported that positive reinforcement in the form of rewards increased tax compliance (Feld, Frey, and Torgler 2006; Torgler 2003).

In contrast, legitimate or soft power is characterized by the legitimacy of the power institutions (Raven, Schwarzwald, and Koslowsky 2003). Subdimensions of legitimate power include the provision of relevant information, the knowledge and skills of the authority, as well as the authority's capacity to make taxpayers identify with its goals and values (Raven et al. 2003). Laboratory experiments suggest that service provision (Alm et al. 2010), that is, information power, has a positive impact on tax compliance. Also, participation in the tax procedure (Wahl, Muehlbacher, and Kirchler 2010; Feld and Frey 2002), that is, power of identification, is shown to promote tax compliance in laboratory experiments. Survey data among taxpayers indicate a positive relation between legitimacy, that is, power of position, provision of relevant information, knowledge and skills, and tax compliance (Gangl et al. 2013; Hartner et al. 2011). Finally, good governance approximating legitimate power is assumed to positively affect taxpayers' willingness to comply with tax laws (Cummings et al. 2009; Smith and Stalans 1991).

### B. The Impact of Coercive Power and Legitimate Power on Psychological Processes and Tax Compliance

The extension of the slippery slope framework (Gangl et al. 2012; Kirchler, Hofmann, and Gangl 2012) assumes that coercive power decreases implicit trust in authorities, which generates an antagonistic climate and enforced compliance by the taxpayers. The basis of implicit trust, on the other hand, is an unconscious reaction generated by shared norms and values, and a sense of social identity. Learning with time that institutions with certain characteristics can be trusted enables implicit trust reactions in the future (Castelfranchi and Falcone 2010). In the tax context, for instance, implicit trust is established through years of good experiences with tax authorities. An antagonistic climate characterizes itself by a “cops and robbers” attitude between the tax authorities and the taxpayers (Kirchler, Hoelzl, and Wahl 2008). The perception is that tax authorities are “cops,” eager to catch tax evaders and punish them, while taxpayers are “robbers,” unwilling to pay taxes and hiding from the authorities. The use of coercive power by the taxation authority breeds suspicion and mistrust. This can result in a vicious cycle where the authorities increase their use of coercion while taxpayers increase their use of evasion or avoidance mechanisms. Ultimately, increased use of evasion by the taxpayer increases the use of coercive powers of the authority, and the cycle continues (Braithwaite 2003; Feld and Frey 2002; Torgler 2002). As a consequence, coercive power is assumed to inhibit implicit trust toward the tax authority and forces taxpayers to pay their taxes; they only pay because they are pressed to do so.

**Figure 1:**
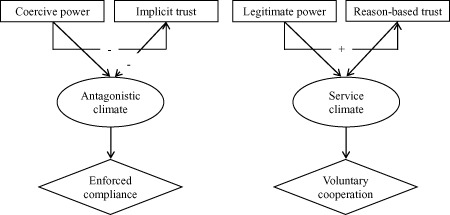
The Impact of Coercive and Legitimate Power on Trust, The Interaction Climate, and the Motivation to Comply.

In contrast, legitimate power is suggested to increase reason-based trust and to stimulate a service climate, and therefore elicit voluntary cooperation by taxpayers. The basis of taxpayers' reason-based trust is rational considerations of the authorities' trustworthiness such as their competence, motivation, or benevolence (Castelfranchi and Falcone 2010). Legitimate power increases reason-based trust, because it provides the taxpayers with reasons to trust such as the authorities' expertise in tax issues and their willingness to share valuable information with the taxpayers. When legitimate power is prevalent in a service climate, it elicits voluntary cooperation. In such a service climate, taxpayers comply with tax law because they believe that authorities will reciprocate with cooperation. The interaction is characterized by clearly defined fair-play rules, and taxpayers' motivation to pay taxes is voluntary because taxpayers perceive paying honestly as the easiest and most hassle free way to handle tax issues. Figure 1 displays the different motivational paths through which coercive power and legitimate power affect tax compliance.

We are confident, that by analyzing coercive power and legitimate power separately, and by distinguishing between different qualities of trust, that is, implicit trust and reason-based trust, we are able to clarify the psychological processes that are responsible for tax compliance. This then, fundamentally provides a contribution to current understandings in taxation research. Moreover, by analyzing the combined effects of both coercive power and legitimate power, we can explain the findings of earlier experiments in which high power and high trustworthiness have produced the highest level of tax compliance (Kogler et al. 2013; Wahl, Kastlunger, and Kirchler, 2010). The findings of these experiments suggest that power and trust are not independent determinants of tax compliance but that it is in fact the interaction between the two that is relevant. We assume that the shape of mutual interaction between power and trust depends on the qualities of that power and trust. Experimental manipulation of high power, in combination with a low degree of trustworthiness on the part of authorities, may be interpreted as coercion, while high power in combination with high trustworthiness is likely to be perceived as legitimate expert power, producing the highest degree of tax compliance. A high degree of trustworthiness among the authorities, combined with low power, may produce some reason-based trust but can also breed suspicion that the authorities are unable to guarantee tax compliance by fellow citizens and are thus unable to combat free riders. Additionally, with the combination of power qualities, we can shed light on the underlying psychological processes that occur with the combination of coercive and legitimate power. Finally, it also allows valuable and new insights into the impact of the current practice of tax authorities to apply coercive power as well as legitimate power.

We conducted two studies to examine the impact of coercive and legitimate power. In Study 1, we investigate two experiments that examine the impact of coercive power and legitimate power, respectively, on intended tax compliance, trust, the underlying motivation to pay taxes, and the perceived interaction climate. Study 2 goes a step further and examines a combination of coercive and legitimate power.

Study 1 proves the impact of coercive power on intended tax compliance (e.g., Hasseldine et al. 2007), but the study especially contributes to tax research by shedding light on the underlying psychological processes. In addition, it backs up findings of the impact of legitimate power on intended tax compliance (Alm and Torgler 2011; Feld and Frey 2007; Wenzel 2002) and again reveals the underlying psychological processes. As pure coercive power or legitimate power are seldom wielded, the effect of combined forms of power is of high theoretical and practical interest.

Study 2 exceeds current tax research by combining both forms of power and examining the combined impact on intended tax compliance. Again, Study 2 is especially valuable because it provides unprecedented insight by identifying the underlying psychological processes. Based on earlier findings, the combination of high coercive power and high legitimate power—which could be perceived as especially high legitimate power—prompts the highest intended tax payments. This, in turn, compares combinations of high coercive power with low legitimate power or high legitimate power combined with low coercive power. Tax authorities wielding high coercive and high legitimate power are legitimized professionals protecting honest taxpayers from the free riders who look to exploit other citizens. Thus, coercive power applies to tax evaders but is not as applicable to oneself. This approach resembles a “carrot and stick” policy (Braithwaite 2001) in which authorities recognize those taxpayers worthy of prosecution and those deserving encouragement and support. If high legitimate power wields low coercive power, we perceive high trustworthiness and expect fairly high intended tax compliance. Moreover, we perceive such authorities as benevolent toward taxpayers but probably without sufficient measures at their disposal to restrain free riders from exploitation. Tax authorities holding high coercive power but low legitimate power are perceived as untrustworthy and, because of the effect of coercion, are unlikely to elicit tax compliance. In such a country, taxpayers would perceive authorities as dictatorial and authoritarian, applying arbitrary measures, threatening taxpayers, or causing fear mongering. The combination of low coercive power and low legitimate power is assumed to produce the lowest degree of intended tax compliance because authorities are perceived as being highly untrustworthy. We see such laissez-faire authorities as incapable of effectively levying and collecting taxes. Study 2 examines whether high coercive power leads to lower implicit trust and to increases in enforced compliance. Further, it investigates whether coercive power leads to a more distinct antagonistic climate and to intentions of higher tax payments than low coercive power. Finally, it examines whether high legitimate power triggers more reason-based trust, increases voluntary cooperation, creates a more distinct service climate, and prompts intentions for higher tax payments than low legitimate power.

Studies 1 and 2 show that coercive and/or legitimate descriptions of tax authorities are sufficient to shape intended tax behavior in a fictitious example and, as such, coercive and legitimate behaviors are unnecessary.

## III. Study 1

Study 1 is comprised of two parts, Study 1a and Study 1b, which together allow us to examine the impact of coercive and legitimate power, respectively. Study 1a investigates whether high coercive power leads to high intended tax compliance, low implicit trust, enforced compliance, and to the perception of an antagonistic interaction climate. Study 1b examines the positive influence of legitimate power on intended tax compliance, reason-based trust, voluntary cooperation, and the perception of a service climate.

### A. Study 1A

#### 1. Participants

A convenience sample of sixty-two taxpayers recruited from acquaintances of university members took part in the study (53 percent female; M[age] = 31.81 years, SD[age] = 12.09, range 18–69). A precondition for participation was that they were obliged to pay income tax; persons without taxable income were not permitted. Most participants held final general qualifications for university entrance (47 percent) or a university degree (47 percent). About three-quarters of participants (74 percent) were employed; an additional 16 percent were self-employed.

#### 2. Procedure and Instrument

We used scenarios in an online questionnaire in which the tax authority of a fictitious country was presented as having either high or low coercive power (Box 1). The fictitious scenario in the experimental questionnaire was necessary to manipulate pure high or low coercive power. Although the experimental design was of lower external validity than if existing authorities were described, it allowed precise testing of the impact of coercive power. Participants could put themselves in the fictitious scenario without reminders of real life conditions. Tax authorities in scenarios with high coercive power, for instance were strict, as opposed to lenient, toward tax evaders. Participants were asked to imagine living in a country in which authorities had either high or low coercive power (1st Government), and then they were asked to imagine experiencing a radical change of government (2nd Government), to one in which the power of the tax authorities was either comparatively expanded or diminished. Although taxpayers very rarely experience such a radical change in their lifetimes, this design allows for the investigation of differences in the perception of power within subjects, reducing error variance and increasing statistical power. The experimental design was a 2 (low coercive power versus high coercive power) × 2 (1st Government versus 2nd Government) repeated measures ANOVA-design.

**Box 1. *Low* and High Coercive Power Scenario**

The sanctions for tax evasion in Tovland are very high [*low*]. If tax evasion is discovered, you [*do not*] have to anticipate severe sanctions. The tax authority is [not] lenient toward tax evaders.

On the contrary, after tax audits, there are major [*minor*] financial rewards for correct tax filing independent of the amount of your income. This considerable [*moderate*] amount is credited to the following year's tax return.

After reading each scenario, participants responded to two items that assessed their intended tax compliance, and eighty-seven statements that assessed constructs of the extension of the slippery slope framework on a 6-point Likert scale (1 = totally disagree to 6 = totally agree). While scales from the TAX-I (Kirchler and Wahl, 2010) were applied for enforced compliance and the perception of an antagonistic climate, respectively, the items used to assess voluntary cooperation were adapted from the scale capitulation described in Braithwaite's (2003) inventory of motivational postures. The items for intended tax compliance coercive power, legitimate power, implicit trust, reason-based trust, and the perception of a service climate were newly developed based on the respective concepts (Gangl et al. 2012; Castelfranchi and Falcone 2010; Raven, Schwarzwald, and Koslowsky 2003). We analyzed all scales with principal component analysis and adapted them if reliability, as measured via Cronbach-α, was low. Based on this analysis, we excluded some items from the scales' coercive power and implicit trust. We present descriptive statistics of the final scales in Table 1.

**Table 1:** Scales for Variables of the Extension of the Slippery Slope Framework for the Coercive Power Experiment (N = 62)

Conditions	1st gov't	2nd gov't	1st gov't	2nd gov't	Cronbach-α
	Low	High	High	Low	
N	30		32		
Scale	M (SD)	M (SD)	M (SD)	M (SD)	
Intended tax compliance	4.13 (1.57)	4.88 (1.35)	5.08 (1.09)	4.14 (1.55)	.83
Implicit trust	2.17 (1.10)	2.23 (1.19)	1.88 (0.98)	1.90 (1.24)	.86
Reason-based trust	3.57 (0.72)	3.70 (0.63)	3.41 (0.63)	3.20 (0.75)	.78
Enforced compliance	2.90 (1.28)	3.99 (1.44)	4.01 (1.36)	2.97 (1.22)	.83
Voluntary cooperation	3.19 (1.01)	3.31 (1.10)	2.93 (1.05)	2.72 (1.32)	.72
Antagonistic climate	3.07 (1.09)	3.42 (0.95)	3.49 (0.96)	3.10 (1.23)	.62
Service climate	3.47 (1.20)	3.55 (1.09)	3.31 (1.07)	2.94 (1.16)	.45

#### 3. Manipulation Check for Low Versus High Coercive Power

To examine the success of the manipulation of coercive power, we conducted a 2 (low coercive power versus high coercive power) × 2 (1st Government versus 2nd Government) repeated measures ANOVA, in which perceived coercive power (Cronbach-α = .76) was the dependent variable. The results revealed a main effect of coercive power manipulation (F(1, 60) = 33.24, p > .001, η^2^ = .36). Neither the sequence of governments (F(1, 60) = 0.43, p = .51) nor the interaction between coercive power and the sequence of governments reached significance (F(1, 60) = 0.04, p = .84). As analyses conducted with additional control groups in which power did not change from the first to the second government have shown, the contrast between high coercive power and low coercive power had no impact on the perception of coercive power. Low coercive power (1st Government: M = 3.41, SD = 1.35; 2nd Government: M = 3.24, SD = 1.25) and high coercive power (1st Government: M = 4.71, SD = 1.05; 2nd Government: M = 4.78, SD = 1.17) were in accordance with the manipulation. After reading the high coercive power scenario, for instance, participants perceived that the tax authorities were more able to cause taxpayers inconvenience and to severely punish tax evaders than participants who had read the low coercive power scenario. When filling in the coercive power items, they were not able to read the scenario text again. This essentially means that we assessed participants' sentiments towards the fictitious tax authorities but not the wording of the scenario text.

#### 4. Results

##### (a) Impact of Low Coercive Power Versus High Coercive Power on Intended Tax Compliance

We investigated the hypothesis that high coercive power leads to higher intended tax compliance. A 2 (low coercive power versus high coercive power) × 2 (1st Government versus 2nd Government) repeated measures ANOVA revealed an effect of coercive power on intended tax compliance (F(1,60) = 20.08, p < .001, η^2^ = .25). There was no significant main effect of the sequence of governments (F(1, 60) = 0.11, p = .74); nor was there an interaction effect (F(1, 60) = 0.25, p = .62). Hence, high coercive power prompts higher intended tax compliance. The respective means are displayed in Table 1.

##### (b) Impact of Low Coercive Power Versus High Coercive Power on Implicit Trust, Enforced Compliance, and Antagonistic Climate

A 2 (low coercive power versus high coercive power) × 2 (1st Government versus 2nd Government) repeated measures MANOVA—with implicit trust, enforced compliance, and the perception of an antagonistic climate as dependent variable —revealed a main effect of coercive power (F(3, 58) = 8.62, p < .001, η^2^ = .31), no significant effect as a result of the sequence of governments (F(3, 58) = 0.49, p = .70) and no interaction effect (F(3, 58) = 0.07, p = .97).

Specifically, the univariate analyses revealed significant main effects of coercive power intensity for enforced compliance (F(1, 60) = 24.98, p < .001, η^2^ = .29) and for the antagonistic climate (F(1, 60) = 5.98, p < .05, η^2^ = .09). However, there was no main effect of coercive power on implicit trust (F(1, 60) = 0.08, p = .83). The respective means are displayed in Table 1.

#### 5. Discussion

Study 1a shows that high coercive power can lead to higher intended tax compliance, feelings of enforced compliance, and to the perception of an antagonistic climate. Implicit trust did not vary with variations in the amount of coercive power. Coercive power influenced intended tax compliance through the predicted processes, with the exception that coercive power had no impact on implicit trust. Thus, Study 1a supports experimental findings that high fines and frequent audits induce higher tax compliance (e.g., Park and Hyun 2003). However, it also shows that the actual manipulation of the amount of fines and the frequency of audits are not crucial, but the simple description of the tax authorities as severely fining and strictly auditing without any numeral specification are crucial. As the impact of described coercive power can be confirmed, the impact of legitimate power, that is, power because of legitimacy of the position of the tax authorities, their knowledge and skills, their capacity to be figures for identification, and their willingness to offer and provide relevant information (Raven, Schwarzwald, and Koslowsky 2003), is still in need of investigation. While earlier studies have covered parts of legitimate power (e.g., service quality [Gangl et al. 2013]), it has not been investigated inclusively.

### B. Study 1B

#### 1. Participants

A convenience sample of seventy-eight taxpayers recruited from acquaintances of university members took part in the study (44 percent female; M[age] = 31.67 years, SD[age] = 10.88, range 18–64). A precondition for participation was again that they had to pay income tax; persons without a taxable income were not permitted. Most participants held final general qualifications for university entrance (42 percent) or a university degree (39 percent). More than half of all participants (53 percent) were employed; an additional 14 percent were self-employed.

#### 2. Procedure and Instrument

The procedure and instrument used in Study 1b were identical to those used in Study 1a except for the scenarios that manipulated high and low degrees of legitimate power. Tax authorities with low legitimate power, as opposed to high legitimate power, were incompetent versus competent professionals (Box 2). The experimental design was a 2 (low legitimate power versus high legitimate power) × 2 (1st Government versus 2nd Government) repeated measure ANOVA-design. We present descriptive statistics of the scales in Table 2.

**Box 1 *Low* and High Legitimate Power Scenario**

The current government was formed after a democratic election, whereby independent observers report a regular [*irregular*] procedure. The government provides the tax authority with a consistently [*inconsistently*] worded law to prosecute tax evaders.

It is known that the tax authority makes [*no*] allowances with taxpayers regarding small errors and is also [*not*] forthcoming at audits.

The tax authority in Tovland proves to be [*little*] efficient. The competence of its employees regarding their advice for taxpayers and the processing of audits is well known [*questioned*].

In addition, the tax authority of Tovland offers ample [*very little*] information to support the preparation of the tax return. Similarly, the audit and sanctions procedure for tax evaders are very transparent [*nontransparent*].

In general, the tax authority of Tovland has a good [*bad*] reputation and is [*not*] respected for its work.

It has been shown that citizens, based on the degree of their cooperation with the tax authority, have great [*little*] influence on the functioning of the tax authority and therefore on the state.

**Table 2:** Scales for Variables of the Extension of the Slippery Slope Framework for the Legitimate Power Experiment (N = 78)

Conditions	1st gov't	2nd gov't	1st gov't	2nd gov't	Cronbach-α
	Low	High	High	Low	
N	42		36		
Scale	M (SD)	M (SD)	M (SD)	M (SD)	
Intended tax compliance	4.38 (1.22)	5.26 (0.89)	4.28 (1.24)	3.83 (1.35)	.67
Implicit trust	1.26 (0.73)	2.51 (1.05)	2.83 (1.47)	1.54 (0.60)	.89
Reason-based trust	2.76 (0.46)	3.64 (0.58)	3.70 (0.58)	2.85 (0.54)	.94
Enforced compliance	3.51 (1.42)	3.40 (1.56)	3.56 (1.27)	3.92 (1.35)	.92
Voluntary cooperation	2.41 (0.82)	4.18 (1.14)	4.06 (1.20)	2.09 (0.85)	.88
Antagonistic climate	4.59 (1.09)	2.33 (0.97)	2.73 (1.25)	4.53 (1.38)	.95
Service climate	2.38 (1.09)	4.48 (1.07)	4.25 (1.08)	2.58 (1.03)	.80

#### 3. Manipulation Check for Low Versus High Legitimate Power

We undertook the manipulation check for legitimate power with a 2 (low legitimate power versus high legitimate power) × 2 (1st Government versus 2nd Government) repeated measures ANOVA, in which perceived legitimate power (Cronbach-α = .93) was the dependent variable. The analysis revealed a main effect of legitimate power (F(1, 76) = 230.31, p < .001, η^2^ = .75), a significant effect as a result of the sequence of governments (F(1, 76) = 6.30, p < .05, η^2^ = .08) and no interaction effect (F(1, 76) = 1.34, p = .25). As analyses conducted with additional control groups in which power did not change from the first to the second government have shown, the contrast between high legitimate power and low legitimate power had an impact on the perception of legitimate power. After a change from high to low legitimate power, participants perceive legitimate power as higher (M = 3.22, SD = 0.61) as compared to that in the scenario in which power was low under both the first and the second government (M = 2.81, SD = 0.61). This indicates that high legitimate power might have continued to have an effect even after the reduction of said power. Nevertheless, the manipulation worked well, as low legitimate power (1st Government: M = 2.85, SD = 0.62; 2nd Government: M = 3.22, SD = 0.61) and high legitimate power (1st Government: M = 4.60, SD = 0.64, 2nd Government: M = 4.45, SD = 0.61) were perceived as such. When we reduced power in the second government, the effect of high legitimate power seemed to continue to have an effect. After reading the high legitimate power scenario, for instance, participants felt that the tax authorities were more efficiently collecting taxes and were more appreciated by taxpayers for providing comprehensive information and advice than participants who had read the low legitimate power scenario. Again, participants were unable to read the scenario text while filling in the items so that we could assess sentiments toward the fictitious tax authorities but not the wording of the scenario text.

#### 4. Results

##### (a) Impact of Low Versus High Legitimate Power on Intended Tax Compliance

We investigated the hypothesis that high legitimate power leads to higher intended tax compliance. Results of a 2 (low legitimate power versus high legitimate power) × 2 (1st Government versus 2nd Government) repeated measures ANOVA revealed that legitimate power (F(1, 76) = 19.49, p < .001, η^2^ = .20) and the sequence of governments (F(1, 76) = 11.97, p = .001, η^2^ = .14) both had significant effects on intended tax compliance. We found no significant interaction effect (F(1, 76) = 2.11, p = .15). High legitimate power led, on average, to higher intentions of tax compliance than did low legitimate power. Intended tax compliance under the second government was lower than under the first. The respective means are displayed in Table 2.

##### (b) Impact of Low Legitimate Power Versus High Legitimate Power on Reason-Based Trust, Voluntary Cooperation, and Service Climate

The results of a 2 (low legitimate power versus high legitimate power) × 2 (1st Government versus 2nd Government) repeated measures MANOVA showed a main effect of legitimate power (F(3, 74) = 71.83, p < .001, η^2^ = .74), no significant main effect as a result of the sequence of governments (F(3, 74) = 1.22, p = .31), and no interaction effect (F(3, 74) = 1.10, p = .35).

Univariate analyses revealed that legitimate power had a significant impact on reason-based trust (F(1, 76) = 118.90, p < .001, η^2^ = .61), voluntary cooperation (F(1, 76) = 175.20, p < .001, η^2^ = .70), and the perceptions of a service climate (F(1, 76) = 126.65, p < .001, η^2^ = .63). The respective means are displayed in Table 1.

#### 5. Discussion

Study 1b shows that high legitimate power resulted in high intended tax compliance, high reason-based trust, high voluntary cooperation, and the perception of a distinct service climate. Legitimate power influences intended tax compliance through the predicted processes. Study 1b backs up findings (e.g., Gangl et al. 2013) that stress supportive procedures of tax authorities facilitate tax compliance. Again, in the current study it was not the legitimate processes but the description of a legitimate tax authority that was necessary to show modifications in taxpayers' behaviors. Nevertheless, in Study 1, coercive power and legitimate power are tested separately. The effects of the two forces in combination are still to be investigated.

## IV. Study 2

Study 2 tests the impact of coercive power and legitimate power combined on intended tax compliance. It was assumed that high coercive power and high legitimate power exercised in combination would generate the highest degree of intended tax compliance because they result in the tax authority being perceived as a legitimate expert power holding ample trustworthiness. Study 2 examines if there are correlating results with Study 1, which distinguishes the separate impact of coercive and legitimate power on trust, the climate between tax authorities and taxpayers, motivations of enforced compliance, and on the levels of voluntary cooperation.

### A. Participants

A convenience sample of 121 taxpayers recruited from acquaintances of university members (50.4 percent females; M[age] = 38.77 years, SD[age] = 12.15, range 20–68) participated in the study. A precondition for participation was that they had to pay income tax; persons without a taxable income were not permitted. Most participants held a university degree (47.9 percent) or final general qualifications for university entrance (28.1 percent). More than half of the participants (61.2 percent) were employed; an additional 38.4 percent were self-employed.

### B. Procedure and Instrument

The procedure and instrument were similar to those in Study 1, except that tax authorities in this exercise held either low coercive power or high coercive power combined with either low legitimate power or high legitimate power. We presented a case study of a fictitious country to participants, which underwent no change from one government to another. Tax authorities holding low coercive power and high legitimate power were lenient toward tax evaders and competent. Tax authorities holding high coercive power and low legitimate power were strict toward tax evaders and incompetent. Tax authorities wielding low coercive power and low legitimate power were lenient toward tax evaders and incompetent. Finally, tax authorities holding high coercive power and high legitimate power were strict toward tax evaders and competent (Box 3). The experiment employed a 2 (low coercive power versus high coercive power) × 2 (low legitimate power versus high legitimate power) design. The questionnaire consisted of seventy-two items that resembled the scales in Study 1, but in order to achieve improved reliability, we changed some items, and as such, reduced the number of total items. We present scale statistics in Table 3.

**Box 3 *Low* and High Coercive Power and *Low* and High Legitimate Power Scenario**

The sanctions for tax evasion in Tovland are very high [*low*]. If tax evasion is discovered, you [*do not*] have to anticipate severe sanctions. The tax authority is [not] lenient toward tax evaders.

On the contrary, after tax audits, there are major [*minor*] financial rewards for correct tax filing independent of the amount of your income. This considerable [*moderate*] amount is credited to the following year's tax return.

The current government was formed after a democratic election, whereby independent observers report a regular [*irregular*] procedure. The government provides the tax authority with a consistently [*inconsistently*] worded law to prosecute tax evaders.

It is known that the tax authority makes [*no*] allowances with taxpayers regarding small errors and is also [*not*] forthcoming at audits.

The tax authority in Tovland proves to be [*little*] efficient. The competence of its employees regarding their advice for taxpayers and the processing of audits is well known [*questioned*].

In addition, the tax authority of Tovland offers ample [*very little*] information to support the preparation of the tax return. Similarly, the audit and sanctions procedure for tax evaders are very transparent [*nontransparent*].

In general, the tax authority of Tovland has a good [*bad*] reputation and is [*not*] respected for its work.

It has been shown that citizens, based on the degree of their cooperation with the tax authority, have great [*little*] influence on the functioning of the tax authority and therefore on the state.

**Table 3:** Perception of Coercive and Legitimate Power for the Combined Power Experiment (N = 121)

Conditions	Low coercive & low legitimate power	Low coercive & high legitimate power	High coercive & low legitimate power	High coercive & high legitimate power	Cronbach-α
N	29	31	31	30	
Scale	M (SD)	M (SD)	M (SD)	M (SD)	
Perceived coercive power	4.30 (1.33)	3.28 (1.16)	4.77 (1.16)	4.78 (0.94)	.67
Perceived legitimate power	3.31 (1.18)	4.79 (0.84)	3.07 (1.03)	4.68 (0.87)	.95

### C. Manipulation Check for Low and High Coercive Power and for Low and High Legitimate Power

For the manipulation check, we conducted a 2 (low coercive power versus high coercive power) × 2 (low legitimate power versus high legitimate power) MANOVA with perceived coercive power and perceived legitimate power as dependent variables. The results of univariate analyses showed a significant main effect of coercive power on the perception of coercive power (F(1, 117) = 22.07, p < .001, η^2^ = .16). Also, the perception of legitimate power corresponded with the manipulation (F(1, 117) = 74.12, p < .001, η^2^ = .39). Regarding the perception of coercive power, there was a weak significant interaction effect of coercive power and legitimate power (F(1, 117) = 5.93, p < .05, η^2^ = .05); there was no interaction effect, however, as it pertains to the perception of legitimate power (F(1, 117) = 0.11, p = .74). The manipulation was successful, as low and high coercive power and low and high legitimate power were each perceived as having been manipulated in the scenarios. Reading a scenario including high coercive power participants, for instance, suggested that the tax authorities were more able to cause taxpayers inconvenience and to severely punish tax evaders than participants who had read a scenario comprising low coercive power. After reading a scenario including high legitimate power, participants felt that tax authorities were collecting taxes more efficiently. Further, they were more appreciated by taxpayers for providing comprehensive information and advice than participants who concentrated on a scenario with low legitimate power. Again, participants had no possibility to read the scenario text while filling in the items so that we could assess sentiments toward the fictitious tax authorities but not the wording of the scenario text.

### D. Results

#### 1. Impact of Coercive Power and Legitimate Power on Intended Tax Compliance

A 2 (low coercive power versus high coercive power) × 2 (low legitimate power versus high legitimate power) ANOVA with intended tax compliance as the dependent variable showed no main effect of coercive power (F(1, 117) = 1.00, p = .32) but did reveal an effect of legitimate power (F(1, 117) = 23.67, p < .001, η^2^ = .17). We found no interaction effect of coercive and legitimate power (F(117, 1) < 0.01, p = .998). The respective means are displayed in Table 4.

**Table 4:** Scales for Constructs of the Extension of the Slippery Slope Framework for the Combined Power Experiment (N = 121)

Conditions	Low coercive & low legitimate power	Low coercive & high legitimate power	High coercive & low legitimate power	High coercive & high legitimate power	Cronbach-α
N	29	31	31	30	
Scale	M (SD)	M (SD)	M (SD)	M (SD)	
Intended tax compliance	3.71 (1.45)	4.90 (1.28)	3.95 (1.67)	5.15 (0.88)	.89
Implicit trust	1.75 (1.17)	1.84 (0.91)	1.48 (0.77)	1.89 (0.96)	.85
Reason-based trust	2.77 (1.20)	4.36 (1.03)	2.64 (0.95)	4.47 (0.87)	.95
Enforced compliance	3.30 (1.21)	2.81 (1.43)	4.43 (1.22)	4.16 (1.44)	.85
Voluntary cooperation	3.14 (1.13)	4.17 (0.77)	3.34 (1.43)	4.39 (0.92)	.58
Antagonistic climate	4.09 (1.24)	2.27 (1.29)	4.52 (1.05)	2.44 (1.33)	.86
Service climate	2.57 (1.36)	4.78 (1.23)	2.49 (1.12)	4.56 (1.37)	.88

#### 2. Impact of Coercive and Legitimate Power on Trust, Motivations, and Climates

A 2 (low coercive power versus high coercive power) × 2 (low legitimate power versus high legitimate power) MANOVA revealed a significant main effect of coercive power (F(4, 114) = 6.78, p < .001, η^2^ = .19) and of legitimate power (F(4, 114) = 24.27, p < .001, η^2^ = .46) but no interaction effect (F(4, 114) = 0.43, p = .78) on the dependent variables implicit trust, reason-based trust, enforced compliance, voluntary cooperation, perception of an antagonistic climate, and perception of a service climate.

The univariate analyses showed no main effects of coercive power (F(1, 117) = 0.37, p = .54) and legitimate power (F(1, 117) = 2.02, p = .16) on implicit trust. For reason-based trust, there was no main effect of coercive power (F(1, 117) < 0.01, p = .95), although a main effect of legitimate power (F(1, 117) = 86.02, p < .001, η^2^ = .42) was found. Coercive power had a main effect on enforced compliance (F(1, 117) = 26.29, p < .001, η^2^ = .18), whereas legitimate power (F(1, 117) = 2.51, p = .12) did not. Similarly, legitimate power affected voluntary cooperation (F(1, 117) = 27.47, p < .001, η^2^ = .19), whereas coercive power (F(1, 117) = 1.14, p = .29) had no effect. There was no impact of coercive power on the perception of an antagonistic climate (F(1, 117) = 1.80, p = .18), but there was an impact related to legitimate power (F(1, 117) = 75.86, p < .001, η^2^ = .39)—high legitimate power led to low perceptions of an antagonistic climate. High legitimate power also led to perceptions of a more distinct service climate (F(1, 117) = 84.93, p < .001, η^2^ = .42), but there was no effect of coercive power (F(1, 117) = 0.45, p = .51). The respective means are displayed in Table 4.

### E. Discussion

Study 2 expands and partly confirms the findings of Study 1. Unlike Study 1a, coercive power had no significant impact on intended tax compliance, but similar to Study 1b, we found legitimate power to have a significant impact. Specifically, high coercive power and high legitimate power combined generated the highest intended tax compliance (significantly higher than the combination of high coercive power and low legitimate power and the combination of low coercive power and low legitimate power [mean differences 95 percent CIs (0.3, 2.1) and (0.5, 2.4)]). Additionally, if high legitimate power and low coercive power were wielded in combination, intended tax compliance was high. Tax authorities holding high coercive power but low legitimate power induced less intended tax compliance. The results indicated that the wielding of coercive and legitimate power in combination could lead to the perception of especially high legitimate expert power and diminished feelings of coercion. This finding supports earlier findings (Kogler et al. 2013; Wahl, Kastlunger, and Kirchler, 2010) that power combined with trustworthiness is producing highest tax contributions.

## V. General Discussion

The aim of the present research was to examine the impact of coercive and legitimate power on intended tax compliance. In the experimental studies presented here, coercive power and legitimate power are shown to affect intended tax compliance, if applied separately (Table 5). However, if we apply both qualities of power in combination, we find legitimate power, but not coercive power, to have an impact on intended tax compliance. Legitimate power seems to be more relevant than coercive power, as predicted by Tyler (2006). Legitimate power and coercive power in combination might be perceived as legitimate expert power, inducing trust by creating the impression that exploitative free riders will be penalized expertly while supporting honest taxpayers in order to elicit the highest intended tax compliance. In cases in which only legitimate power is applied, authorities are perceived as benevolent but without sufficient measures at their disposal to prosecute free riders. Their measures are based on a “toothless theory” (Ariel 2011, 39) inducing some intended tax compliance but at a lesser degree than when exercising the two qualities of power in combination. Authoritarian leadership—in this scenario, the application of only coercive power—resulted in lower intended tax compliance. On the other hand, laissez-faire authorities that wield neither coercive power nor legitimate power are incapable of levying taxes and so generate the least intended tax compliance.

**Table 5:** Summary of Results for Coercive Power, Legitimate Power, and Combined Power Experiments (Study 1a, 1b and 2)

Study	1a	1b	2	
	*Coercive power*	*Legitimate power*	*Coercive power*	*Legitimate power*
Intended tax compliance	+	+	0	+
Implicit trust	0		0	0
Reason-based trust		+	0	+
Enforced compliance	+		+	0
Voluntary cooperation		+	0	+
Antagonistic climate	+		0	−
Service climate		+	0	+

*Note:* + … significant mean differences, whereby high power determined higher means than low

power; − … significant mean differences, whereby high power determined lower means than low

power; 0 … no significant mean differences.

A secondary aim of this research was to investigate the underlying psychological processes of coercive power and of legitimate power and their influence on trust, motivations to comply, and the perceived climate. Coercive power was not seen to have had the expected negative impact on implicit trust in either study (cf. Nooteboom 2002; Kramer 1999) or the predicted positive effect on the antagonistic climate in Study 2, but its effects were in line with the assumptions regarding enforced compliance (Table 5). The absence of a negative impact of coercive power on implicit trust could stem from a manipulation and an assessment problem. As implicit trust is a concept that develops over time, both as an automatic and a learning process, the authorities described in the fictitious scenarios might not have been able to establish or destroy implicit trust. In future research, scenarios should take into account the fact that the development of implicit trust requires positive past experiences with tax authorities. The absence of an impact of coercive power on perceptions of an antagonistic climate and the fact that legitimate power effected this perception negatively in Study 2 suggest that the climate is not stimulated by coercive power but is, in fact, inhibited by legitimate power. This is also supported by the results of Study 1b, which show that high legitimate power led to significantly lower perceptions of an antagonistic climate (F(1, 60) = 5.98, p < .05, η^2^ = .09; note that coercive power was not manipulated in this study). Again, the reason could be that as soon as legitimate power is wielded, coercive power and legitimate power exercised in combination are perceived as legitimate expert power and thereby act to reduce any potential feelings of coercion. Future research should seek to investigate and confirm this hypothesis.

As predicted, legitimate power affected reason-based trust in both studies, supporting the earlier finding that legitimate sanction systems stimulate trust (Mulder et al. 2006). We also confirm in both studies that legitimate power has a positive impact on voluntary cooperation and on perceptions of a service climate. For the impact of legitimate power, the assumptions are confirmed; however, assumptions surrounding the impact of coercive power require modification. The perceptions of coercive power and the respective behavioral intentions are dependent on the presence of legitimate power.

In addition to its merits, this article also has certain limitations. The recruitment of the participants for the studies—convenience samples of acquaintances of university members—might have led to a specific selection effect. Participants were, on average, better educated than the general population. However, the studies present first theoretical confirmations; additional research with different samples will have to follow. As with all laboratory experiments working with scenarios, the external validity of the presented studies is lower than with, for instance, field experiments. Nevertheless, conducting a field experiment manipulating low and high coercive and/or legitimate power seems impossible. For example, punishing some taxpayers, while others knowingly get away with tax evasion, or granting some taxpayers support, while neglecting others, seems a highly unlikely behavior of tax authorities. Furthermore, in such a field experiment, other influencing factors, such as regional specifics, could not be kept constant. The present experimental design using scenarios only allows specific investigation of the impact of the forms of power, systematically holding other factors stable. In future experiments, this needs to be extended; in the laboratory, not only intended but actual experimental tax behavior should be assessed. Another area for future research will be not only to describe coercive and/or legitimate tax authorities but to let participants experience coercive and/or legitimate power of tax authorities. It can be argued that, in both Study 1 and Study 2, the descriptions of coercive and legitimate power differ slightly: the coercive power scenario is a bit shorter than the legitimate power scenario. In future studies, coercive power and legitimate power will have to be phrased comparably, investigating whether researchers can successfully replicate the results of Study 2. However, the current results are plausible, support the assumptions of Tyler (2006), and back up existing findings (e.g. Kogler et al. 2013; Kogler, Muehlbacher, and Kirchler 2011; Muehlbacher, Kirchler, and Schwarzenberger 2011; Muehlbacher and Kirchler 2010; Wahl, Kastlunger, and Kirchler, 2010). These findings show that legitimate power, with both high and low degrees of coercive power, is more effective at inducing tax compliance than is coercive power with low legitimate power.

For practitioners, the results of Study 1 and Study 2 are interesting and applicable to their work. They show that coercive power and legitimate power have an impact on intended tax compliance, but if exercised in combination, legitimate power is able to alter perceptions of coercive power. Authorities should concentrate on strategies of good governance while simultaneously seeking to protect honest taxpayers from tax evaders. Based on theoretical assumptions, they would establish a service climate, reduce antagonistic interactions, and increase voluntary cooperation, as to secure tax revenues and characterize the societal climate by good and respectful relations between tax authorities and taxpayers. Neither authoritarian leadership nor good governance alone would be able to produce these results. Authoritarian leadership would generate less tax revenues and a climate of mistrust in society; good governance would contribute to a friendly climate but could not deliver comparable tax revenues. This finding strongly supports the responsive regulation approach (Braithwaite 2001), which argues that taxpayers need to be addressed differently depending to the social distance between them and authorities.

Based on the findings, we can say that supportive procedures should have an impact on taxpayers' trust in tax authorities and that coercion is important as long as it directs toward free riders. If coercion or supportive procedures are applied in isolation, some tax revenues could be secured, but if the two qualities of power were applied in combination, increasing and voluntary contributions could be expected.
